# Euthermic Endocarditis

**DOI:** 10.1371/journal.pone.0080144

**Published:** 2013-11-11

**Authors:** Daniel C. DeSimone, Larry M. Baddour, Brian D. Lahr, Heath H. Chung, Walter R. Wilson, James M. Steckelberg, for the Mayo Cardiovascular Infections Study Group

**Affiliations:** 1 Infectious Diseases, Mayo Clinic College of Medicine, Rochester, Minnesota, United States of America; 2 Biomedical Statistics and Informatics, Mayo Clinic, Rochester, Minnesota, United States of America; 3 Infectious Diseases, John A. Burns School of Medicine, University of Hawaii, Handicap International, Honolulu, Hawaii, United States of America; King’s College London School of Medicine, United Kingdom

## Abstract

**Background:**

Most patients with infective endocarditis (IE) manifest fever. Comparison of endocarditis patients with and without fever, and whether the lack of fever in IE is a marker for poorer outcomes, such as demonstrated in other severe infectious diseases, have not been defined.

**Methods and Results:**

Cases from the Mayo Clinic, Rochester, Minnesota, Division of Infectious Diseases IE registry, a single-center database that contains all cases of IE treated at our center. Diagnosis date between 1970 and 2006, which met the modified Duke criteria for definite endocarditis, without fever was included. There were 240 euthermic endocarditis cases included in this analysis, with 282 febrile controls selected by frequency matching on gender and decade of diagnosis. Euthermic patients had a median age of 63.6 years (±16.1) as compared to 59.0 years (±16.4) in the febrile control group (p=0.001). Median (IQR) symptom duration prior to diagnosis was 4.0 (1.0, 12.0) weeks in the euthermic group compared to 3.0 (1.0, 8.0) weeks in the febrile controls (p= 0.006). From unadjusted analyses, survival rates were 87% in euthermic cases versus 83% in febrile controls across 28-day follow-up (p=0.164), and 72% in euthermic group cases versus 69% in febrile controls across 1-year follow-up (p=0.345). Also unadjusted, the 1-year cumulative incidence rate of valve surgery was higher in euthermic cases versus febrile controls (50% vs. 39%, p= 0.004).

**Conclusions:**

Patients with euthermic endocarditis are older, and lack of fever was associated with longer symptom duration and delayed diagnosis prior to IE diagnosis. Despite a higher unadjusted rate of valve surgery in euthermic patients, the result was not significant when adjusting for baseline confounders. Differences in survival rates at both 28-days and 365-days were not statistically significant between the two groups.

## Introduction

Fever is a complex physiological response to infection, inflammation, and tissue injury which many organisms have the ability to produce by altering their internal thermostat[1]. Fever has been shown to enhance antibody production, T-cell activation, production of cytokines, and neutrophil and macrophage function[2]. Retrospective studies in humans support the notion that a febrile response increases survival of patients with severe infections[3–5]. Patients who fail to develop a fever have a significantly higher mortality than febrile septic patients[2,6,7]. The majority of patients with IE manifest fever and the presence of fever is a minor criterion in the Duke criteria for diagnosis[8]. Whether the subset of patients with IE lacking fever differ in important clinical characteristics and how diagnosis and prognosis are affected, remains undefined to date.

It is conceivable for example, that patients with IE lacking fever, so-called “euthermic endocarditis,” could be subject to a delay in diagnosis and initiation of appropriate antimicrobial and/or surgical therapy, resulting in an increased risk of IE-related complications and poorer outcomes. To evaluate the effects of fever, or lack thereof, on disease prognosis, we performed a retrospective cohort study that included patients with IE seen at Mayo Clinic between 1970 and 2006. A cohort of patients with “euthermic” endocarditis were identified, and compared to a frequency matched sample of endocarditis patients with fever. Clinical characteristics and outcomes were characterized and compared by defined outcome-related endpoints.

## Methods

Patients with endocarditis were identified using a combination of electronic resources including the prospectively maintained Mayo Clinic Division of Infectious Diseases endocarditis registry, and institutional electronic medical and surgical diagnostic indexes. Ethics committee approval was not needed. It was not specifically waived. Written consent was given by the patients for their information to be stored in the hospital database and used for research. The data was de-identified immediately after data was collected from the medical record.

### Case Selection

Included patients were 18 years of age or older at the time of diagnosis and met criteria for definite IE according to modified Duke criteria[8] between 1970 through 2006. Patients were excluded if they were diagnosed elsewhere with no diagnostic studies available in medical records, if they declined research authorization, or if there were no temperature measurements recorded in the patient’s medical record. Overall, 240 euthermic IE patients were identified and included in our analysis. There were 287 febrile controls selected by frequency matching on gender and decade of diagnosis**.**

### Definitions

*Fever* was defined as one or more documented measured core temperatures >38.0° C (100.4° F). *Euthermic* endocarditis included patients who have all documented temperatures ≤38.0° C (100.4° F). *Symptom duration* is the time from patient reported onset of symptoms to the date of diagnosis. *Diagnosis date* is the date diagnostic criteria were first met, typically of the date of first positive blood cultures, or the date parenteral antibiotic therapy was initiated in those with negative blood cultures. Indication and date of *cardiac valve surgery* were recorded if valve surgery occurred at any point after the diagnosis.

### Data collection

Relevant inpatient and outpatient medical records of all patients were reviewed by the investigators. Medical history and clinical data were obtained by thorough review of the entire medical record, including daily physicians’ progress notes and all subspecialty consultations. A standardized case report form with the following variables was developed and included demographic, clinical, laboratory, microbiology, and outcome data (date of death or date of last follow up), date of IE diagnosis, date of onset of symptoms, symptom duration, echocardiographic evidence, need for surgical intervention, immunocompromise (if any), and use of antipyretics at the time of diagnosis including the type and dosage were recorded (Acetaminophen, systemic steroids, NSAIDS including ASA greater than or equal to 325mg, selective COX-2 inhibitors). 

### Statistical analysis

Descriptive statistics on baseline variables are presented as median (interquartile range [IQR]), mean (standard deviation [SD]) or count (percentage) as appropriate. Clinical characteristics were formally compared between febrile and afebrile patients using the Student’s t test or Wilcoxon rank-sum test for continuous data, and the chi-square test for categorical data. 

For patient outcomes following IE diagnosis such as all-cause mortality and valve replacement, the Kaplan-Meier method was used to estimate 28-day and 365-day survival (event-free) rates, and both the log-rank test and Cox proportional hazards (PH) regression models were used to assess the association of the outcome with euthermic endocarditis. Since the two groups under comparison were unmatched, a multiple variable Cox PH model was fit for each endpoint to control for baseline differences between groups, and an adjusted hazard ratio (HR) and 95% confidence interval (CI) was estimated to summarize the risk of all-cause mortality (or valve replacement) in euthermic subjects relative to the those who were febrile. All analyses were carried out with the statistical software package SAS, version 9.2 (SAS Institute, Cary, NC, USA).

## Results

Two hundred forty euthermic patients were identified (20% of IE registry patients) and were included in our analysis. Euthermic patients had a mean (±SD) age of 63.6 (±16.1) years as compared to 59.0 (±16.4) years in the febrile control group (p=0.001). Median (IQR) symptom duration prior to diagnosis was 4.0 (1.0, 12.0) weeks in the euthermic group compared to 3.0 (1.0, 8.0) weeks in the febrile controls (p=0.006) (Table 1).

**Table 1:** Comparison of Baseline Descriptives and Follow-Up Outcomes between Euthermic vs. Febrile Groups.

**Variable**	**Total (n=522)**	**Euthermic (n=240)**	**Febrile (n=282)**	**P-value**
*Baseline Characteristics*				
Age at Diagnosis	61.1±16.4	63.6±16.1	59.0±16.4	0.001
Symptom Duration^	4.0 (1.0, 12.0)	4.0 (1.0, 12.0)	3.0 (1.0, 8.0)	0.006
Rx Length^	30.0 (17.0, 44.0)	31.0 (16.0, 46.0)	30.0 (17.0, 43.0)	0.646
Prior Antibiotics:				0.095
. No	292 (56%)	122 (51%)	170 (60%)	
. Yes	158 (30%)	81 (34%)	77 (27%)	
. Unknown	72 (14%)	37 (15%)	35 (12%)	
Diabetes Mellitus	75 (14%)	39 (16%)	36 (13%)	0.258
Hemodialysis	38 (7%)	21 (9%)	17 (6%)	0.233
Immunocompromised	70 (13%)	40 (17%)	30 (11%)	0.044
Abscess cardiac:				0.101
. No	446 (85%)	201 (84%)	245 (87%)	
. Yes	62 (12%)	35 (15%)	27 (10%)	
. Unknown	14 (3%)	4 (2%)	10 (4%)	
Antipyretic	292 (56%)	74 (31%)	218 (78%)	<.001
Organism:				<.001
. Staph Aureus	116 (22%)	43 (18%)	73 (26%)	
. Enterococcus spe	62 (12%)	36 (15%)	26 (9%)	
. Coag Neg Staph	60 (11%)	34 (14%)	26 (9%)	
. Culture Neg	20 (4%)	16 (7%)	4 (1%)	
. Other	116 (22%)	52 (22%)	64 (23%)	
. Viridans Strep	142 (27%)	54 (23%)	88 (31%)	
. Unknown	6 (1%)	5 (2%)	1 (0%)	
*Follow-Up Outcomes*				
All-cause mortality+				
. 28 days following diagnosis	78 (85%)	30 (87%)	48 (83%)	0.164 (0.260*)
. 1 year following diagnosis	146 (71%)	63 (72%)	83 (69%)	0.345 (0.109*)
Valve replacement+				
. 28 days following diagnosis	150 (31%)	85 (38%)	65 (25%)	0.001 (0.138*)
. 1 year following diagnosis	202 (44%)	108 (50%)	94 (39%)	0.004 (0.546*)

^ Median (Q1, Q3); p-value obtained from Wilcoxon rank sum test

+ Cumulative incidence: # events (Kaplan-Meier event rate, %); p-value obtained from log-rank test

* p-value from multivariable Cox PH regression model adjusting for baseline group differences

^ Median (Q1, Q3); K-W rank sum test

+ # deaths (Kaplan-Meier survival %); log-rank test

There was a significant association between euthermic endocarditis and the type of causative microorganisms isolated from blood cultures (p<0.001) as shown in Table 1. In particular, patients with IE caused by viridans group streptococci or *S. aureus* tended more likely to be febrile while patients with culture-negative IE were more likely to be euthermic.

The rates of individual host factors, including diabetes mellitus and chronic hemodialysis, were not statistically different between the two groups. However, there was a significant association between euthermic endocarditis and being immunocompromised (p=0.044), as shown in Table 1.

**Figure 1:**
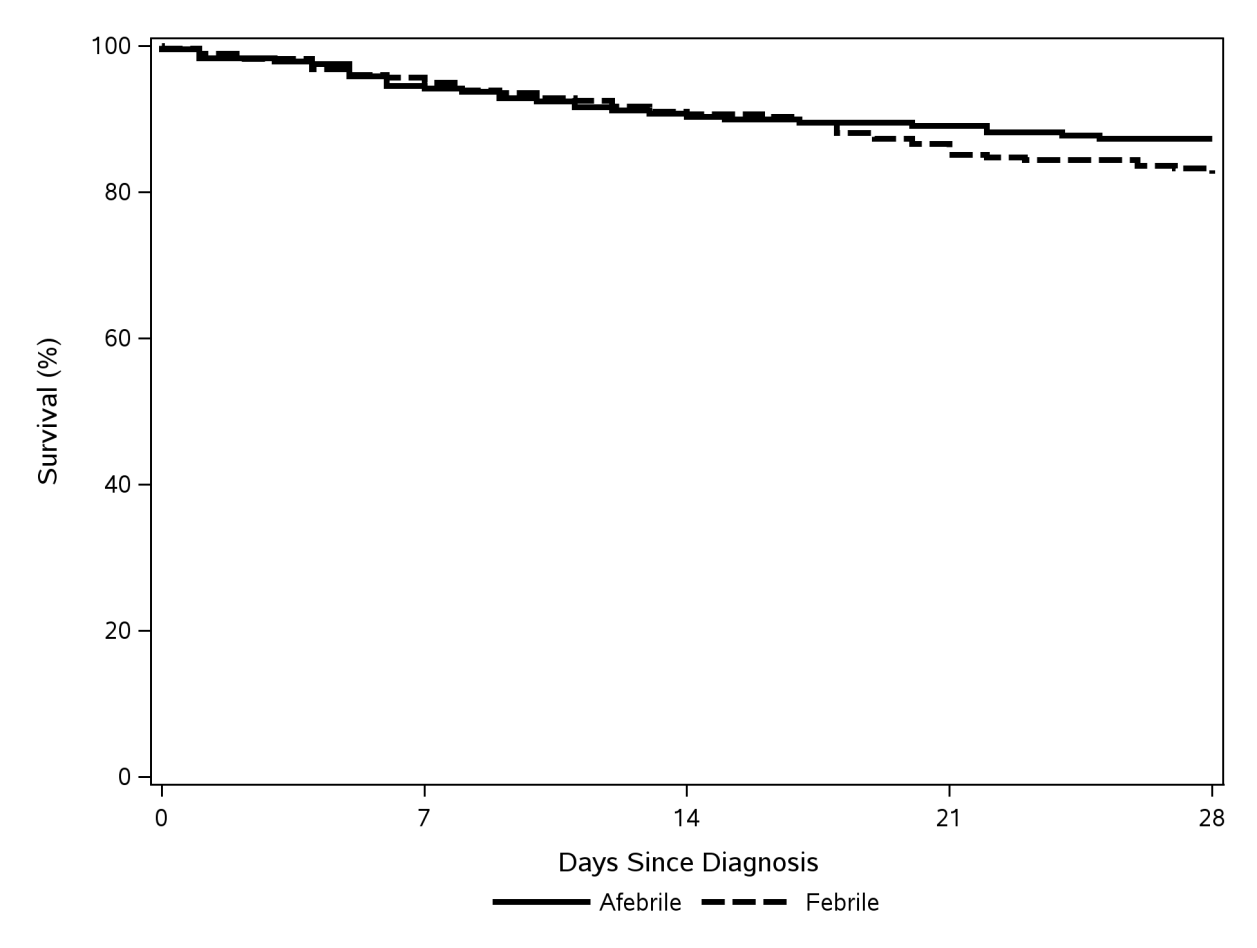
28-day survival.

**Figure 2:**
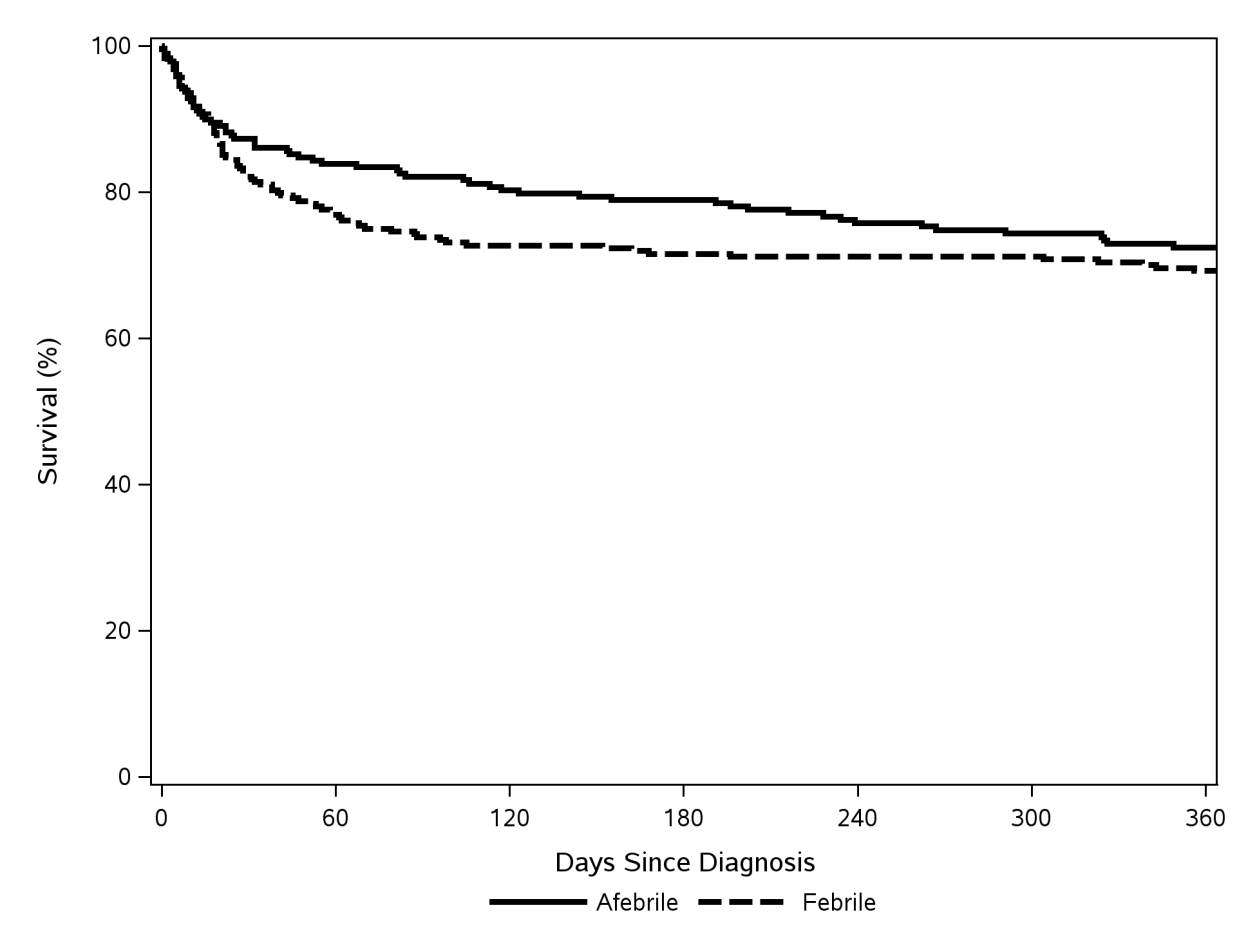
365-day survival.

Based on unadjusted survival analysis comparing the two groups, there were no significant differences in mortality across 28-days (p= 0.164) or 365-days (p= 0.345) of follow-up (Figures 1 and 2). Controlling for baseline differences between groups, the risk of all-cause mortality was also not significantly different between euthermic cases and febrile controls over 28 days (HR=0.72; 95% CI, 0.41-1.27; p=0.260) or 365 days (HR=0.71; 95% CI, 0.47-1.08; p=0.109) of follow-up.

Based on unadjusted cumulative incidence across 1-year follow-up, rates of valve surgery were higher in patients who were euthermic compared to those who were febrile (50% vs. 39%, p= 0.004). However, adjusting for baseline differences between groups, there was no significant association between euthermic endocarditis and having valve replacement (HR=1.11; 95% CI, 0.79-1.58; p=0.546).

## Discussion

 To our knowledge this study is the first to describe the characteristics and outcomes of “euthermic endocarditis” patients, and compare these to febrile endocarditis patients. Patients with euthermic endocarditis were older, had a longer duration of symptoms prior to diagnosis, and required valve surgery at a higher rate than did matched febrile control patients. There was no statistically significant difference in the rates of diabetes mellitus or chronic hemodialysis between the two groups, but there was a significant association between euthermic endocarditis and being immunocompromised (p=0.044).

The diagnosis of IE involves a constellation of signs, symptoms, laboratory, radiologic and echocardiographic findings. In patients presenting with few or none of the classic Oslerian manifestations of bacteremia, fever, peripheral emboli, and immunologic vascular phenomena, the diagnosis of IE is challenging[9]. This potentially could delay initiation of therapy and impact IE-associated morbidity and mortality. Autopsy findings[10] support this notion among patients without a premorbid diagnosis of IE.

Fever prevalence in IE patients has ranged from 47% to 96% in different surveys[11–13]. Fever not only plays a role in the host immune response to infection, but can also serve as a key indicator to an underlying infectious process. Ortega et al., evaluated over 1600 community-acquired bacteremia adult patients and found that the lack of fever at the time of bacteremia was an independent predictor of increased mortality[14]. Patients who fail to mount a febrile response may have an immune deficit, may have advanced age, or may be infected with organisms that are less likely to cause fever, as demonstrated in one report where only 5 out of 22 patients with Q fever endocarditis had fever[15]. Q fever endocarditis, however, is not likely to account for the findings of euthermic endocarditis in our investigation due to the rarity of this syndrome in patients seen at our medical center[16].

The immune system declines with age, so-called immunosenescence, and this can predispose to development of infections[17,18]. Advanced age with dysregulated immune function can predispose to infection and may affect survival rates at 28-days and 365-days. Despite progress in diagnostic and therapeutic accuracy, almost half of IE episodes have at least one complication[15,19] and earlier diagnosis and proper medical therapy may result in fewer complications. Advanced age is associated with worse clinical outcomes in IE due to less pronounced clinical symptoms of disease as compared to those in younger patients[20,21]. In a prospective observational cohort study, clinical evidence of IE was found less often in elderly patients than in younger patients such as embolic events, Osler nodes, Roth spots, Janeway lesions, and conjunctival hemorrhages[22]. Reduced frequency of fever and leukocytosis seen in older patients with IE can reduce clinical suspicion and delay diagnosis[23].

 The presence of fever is likely dependent on an assemblage of host factors and the infecting pathogen. The host response to microbial infection is mediated by the release of inflammatory substances, activated product C3a, interleukin 6, and phospholipase A2, which may help the host to eradicate the invading organisms[24]. In the work presented herein, *S. aureus* and viridans group streptococci were identified more often in febrile patients. In those who had culture-negative results, they were more likely be afebrile (p= 0.001). *S. aureus* produce numerous toxins such as 33-kd protein-alpha toxin that cause pore formation and induce proinflammatory changes in mammalian cells, as well as the pyrogenic-toxin super-antigens that bind to major histocompatibility complex class II proteins, which cause T-cell proliferation and cytokine release, resulting in fever, hypotension, capillary leak, disseminated intravascular coagulopathy, and multiorgan dysfunction[25]. 

Chu, et al. concluded that factors indicative of the host-pathogen interaction in IE (diabetes mellitus, *S. aureus* infection, acute physiological severity, and embolic events) are early independent determinants of in-hospital death[26]. Chu did not further characterize “acute physiology” where APACHE II (Acute Physiology, Age, Chronic Health Evaluation) score at the time of presentation was assessed; body temperature is one of the components of this scoring system. Fever or the lack of fever may be an independent early predictor of increased complications or mortality in IE.

Our study has limitations. This is a single center design with a large portion of cases of IE referred to Mayo for surgical intervention and therefore may introduce a referral bias towards a higher rate of surgical intervention. This should however hold true for both patient groups as we do not expect a bias toward febrile vs. euthermic patients. Our study period extends through 2006; although more recent patient data have not been collected, we believe that findings in this cohort are likely reflective of more recent cases as diagnostic procedures in IE have not appreciably changed over the past six years.

The definition of fever is arbitrary. In some literature, fever is defined as a core temperature of >38.0°C (100.4°F), whereas in other sources, fever is defined as two consecutive temperature elevations to >38.3°C (101°F). Normal body temperature is generally considered to be 37.0°C (98.6°F) with a circadian variation between 0.5 to 1.0°C[2]. We used the aforementioned definition of >38.0°C as this was the definition originally used in the Duke criteria[8]. In multivariable Cox PH regression for estimating the risk of all-cause mortality in euthermic vs. febrile patients, the overall number of deaths across 28 follow-up days provided sufficient power to adjust for several baseline group differences, but not additionally for risk factors associated with mortality.

## Conclusions

To our knowledge, this is the first study that focuses on patients with euthermic endocarditis. Compared to febrile controls (unadjusted for other variables), euthermic patients were older and had longer symptom duration prior to IE diagnosis, and had a higher rate of valve surgery (although this result did not remain significant when controlling for group differences). Differences in survival rates at both 28-days and 365-days were not statistically significant between the two groups, either unadjusted or adjusted for baseline differences. Antipyretic use was uncommon among the euthermic patients and suggests that host-pathogen interactions are likely operative in the lack of production of fever in these patients. A better understanding of the pathogenesis and clinical appreciation of euthermic endocarditis is needed to facilitate an earlier diagnosis and timely initiation of antimicrobial therapy to potentially improve outcomes.
